# Role of Carbamazepine in the Symptomatic Treatment of Subacute Sclerosing Panencephalitis: A Case Report and Review of the Literature

**DOI:** 10.1155/2013/327647

**Published:** 2013-03-07

**Authors:** Sandhya Ravikumar, John Ross Crawford

**Affiliations:** ^1^University of California, San Diego School of Medicine, 9500 Gilman Drive, La Jolla, CA 92093, USA; ^2^Division of Child Neurology, Department of Neurosciences and Pediatrics, Rady Children's Hospital, University of California, San Diego, 8010 Frost Street, Suite 400, San Diego, CA 92123, USA

## Abstract

We describe the clinical presentation and clinical course of subacute sclerosing panencephalitis in a 13-year-old previously healthy boy who recently immigrated to the United States from Iraq. He presented with macular retinopathy, followed by progressive myoclonus and encephalopathy. After extensive workup, a diagnosis of subacute sclerosing panencephalitis was suspected by the presence of period epileptiform discharges on electroencephalogram and confirmed by elevated measles titers in the cerebrospinal fluid. Combination immunomodulatory therapy with isoprinosine, ribavirin, and intra-Ommaya interferon alpha did not result in clinical improvement. Within days following the administration of carbamazepine, there was remarkable improvement in the myoclonus and he was able to ambulate independently for a period of 4 months at which time he unfortunately progressed to a vegetative state. This case highlights the importance of carbamazepine as a potential first line symptomatic treatment of subacute sclerosing panencephalitis and provides a review of the literature on the subject.

## 1. Introduction

Subacute sclerosing panencephalitis (SSPE) is a progressive neurodegenerative disease related to a prior infection with measles virus. The pathogenesis is thought to be due to a mutation in the measles virus protein that allows spread to the brain [1]. Patients typically have the primary measles at less than two years of age, followed by a latent period of 6 to 8 years, after which the SSPE symptoms manifest, although SSPE has also been documented in adults [2]. While the clinical presentation is variable, documented stages include (1) behavioral and cognitive disturbances, followed by (2) impairment of speech and development of myoclonus then (3) loss of speech and difficulty with ambulation, and weight loss, followed by (4) persistent vegetative state [3]. According to Dyken [4], pathologically, the virus initially has its effects on the cortex, followed by the basal ganglia, then the brainstem, which mirror the stages of the disease. After the onset of clinical symptoms, death usually occurs within 1 to 3 years. On autopsy, pathology in late stages typically shows widespread degeneration of neurons and gross structures, vascular lymphocytes throughout the white matter and brainstem, and intranuclear inclusions in neurons and oligodendrocytes [2, 5]. 

Diagnosis is usually made via elevated measles antibody titers in the blood and CSF. The CSF titers may be initially within the normal range for the first few months of the disease. While neuroimaging could appear normal in stages 1 and 2, magnetic resonance imaging (MRI) findings characteristically show cerebral atrophy, white matter abnormalities, and signal change in the basal ganglia [6]. The most common finding on electroencephalography (EEG) is a generalized periodic spike and waves occurring every 5–10 seconds in conjunction with myoclonic episodes [2].

The treatment of SSPE is nonuniform. Alpha interferon has been shown to recover loss of function, but more commonly it stabilizes the disease up to several years [7]. Other treatments include beta interferon and isoprinosine, which have had variable success [8]. Combination treatments with alpha or beta interferon and isoprinosine have demonstrated better outcomes [9–11], but ultimately, the disease remains progressive and fatal. For treatment of myoclonus, case reports have shown carbamazepine to be more effective than antiepileptics such as clonazepam and valproic acid in improving myoclonus [12–16]. 

We report a case of SSPE with marked, although transient, improvement in myoclonus and cognition with carbamazepine despite treatment failures on a wide variety of anticonvulsant, and immunomodulatory therapies. The case highlights the importance of carbamazepine in clinical improvements without altering the long-term clinical or radiographic progression of this rare neurodegenerative disease. 

## 2. Case Presentation

A 13-year-old previously healthy Iraqi-born unvaccinated immigrant boy presented with a one-year history of worsening vision loss. Over the course of 2 months, he developed myoclonic jerks involving all extremities that increased in frequency over the next few months, occurring every 10 seconds. In addition, he had atonic episodes resulting in him falling to the floor. He had difficulty with fine motor movements, including writing and putting on clothes. Over several months, he progressed to being unable to ambulate independently. He had frequent urinary incontinence. His memory and performance in school began to decline, first gradually and then more rapidly after 6 months. He started to have aggressive outbursts towards his family with behaviors such as hitting and biting. His mother reported a febrile illness associated with a rash as a toddler without confirmation of measles.

Initial exam demonstrated decreased visual acuity in the right eye (20/200) and left eye (20/40) and frequent myoclonic jerks. He had evidence of macular retinopathy on fundoscopic evaluation. On mental status examination, he showed progressive impairment with simple multiplication and difficulty with recall and comprehension. He had difficulty with orientation questions. On cranial nerve examination, he had sluggish poorly reactive pupils to direct light bilaterally with normal reactivity on convergence and an oculomotor apraxia. He had diffuse spasticity of both upper and lower extremities with diffuse hyperreflexia. Coordination exam revealed a progressive resting and action tremor bilaterally and an unsteady, wide-based gait.

**Figure 1:**
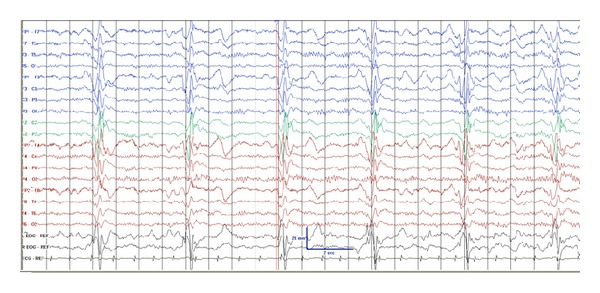
Electroencephalogram showing periodic epileptiform complexes consistent with SSPE.

EEG showed significant frontal slowing during the awake state as well as recurrent periodic generalized periodic epileptiform discharges with preservation of his posterior basic rhythm (Figure 1) that raised suspicion for a diagnosis of a progressive myoclonic epilepsy included in the differential diagnosis: baltic myoclonic epilepsy, Lafora body disease, Unverricht-Lundborg disease, neuronal ceroid lipofuscinosis, sialidosis, myoclonic epilepsy with ragged red fibers, and SSPE.

Following an extensive negative metabolic and infectious workup of serum and CSF samples, the diagnosis of subacute sclerosing panencephalitis was confirmed by elevated serum measles IgG (22.4) and CSF measles IgG (12.0) titers.

## 3. Treatment

The patient was started on inosine and ribavirin, which was continued for several months with no response. He was started on a treatment of Interferon alpha started at 1 million units and increased to 1.5 million units via an intraventricular Ommaya reservoir biweekly for 6 months. This had no effect on his myoclonic jerks or cognitive impairment. The Ommaya therapy was discontinued due to clinical progression. 

Initial anticonvulsants therapy with levetiracetam and clonazepam resulted in minimal improvement of myoclonic seizures and resulted in increased sedation. Similarly, he was refractory to zonisamide and valproic acid. However, it was treatment with carbamazepine monotherapy following discontinuation of the valproic acid that resulted in the most striking clinical improvement.

**Figure 2:**
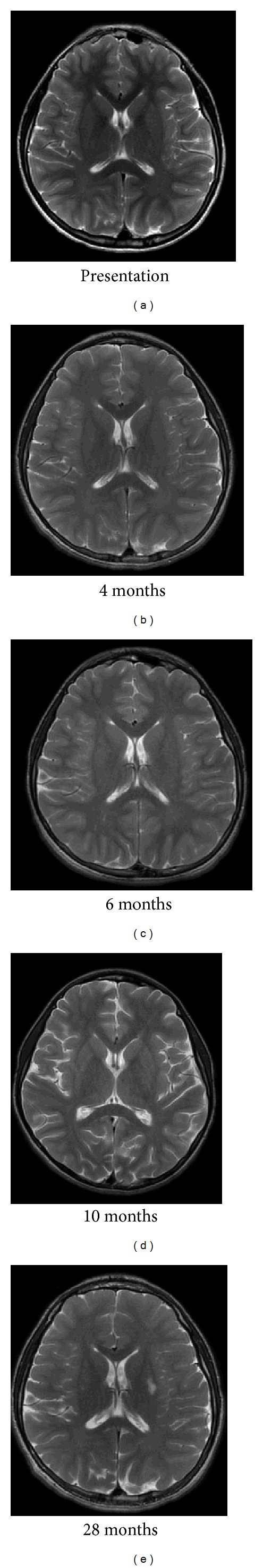
Time course of SSPE by MRI reveals progressive cortical and subcortical white matter volume loss resulting in enlargement of the lateral ventricles.

**Figure 3:**
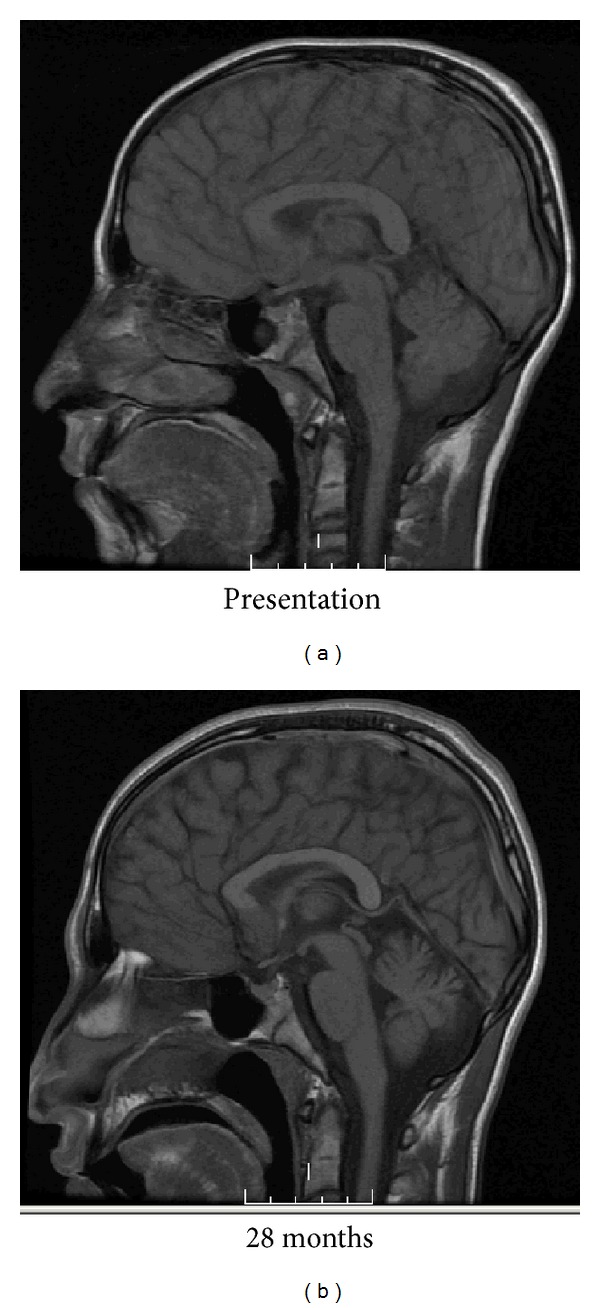
MRI demonstrates progressive atrophy of the corpus callosum, cortex, and cerebellum more than 2 years after the diagnosis of SSPE.

The duration and frequency of the episodes of the myoclonic jerks significantly shortened, and the number of atonic seizures diminished. He was able to ambulate again without assistance. His mental status and cognition improved, and he was able to go to school again. His neurological exam showed improvement of oculomotor apraxia and his urinary incontinence improved. Unfortunately, these astounding clinical effects diminished after several months, after which, in spite of increasing dosages and stable carbamazepine levels, he showed progressive deterioration. These clinical changes correlated MRI changes noted over the course of his disease, as evidenced by diffuse volume loss of the periventricular white matter (Figure 2), corpus callosum, and cerebellum (Figure 3). After several months of improvement, he had an increase of his myoclonus frequency and was progressively unable to ambulate or interact with his environment and now remains in a vegetative state.

## 4. Discussion

Our patient presented with symptoms, CSF studies, MRI brain imaging, and EEG findings consistent with SSPE. Clinically, the patient appeared to be at Stage 2 according to the Jabbour stages, which is characterized predominantly by myoclonic jerks in addition to mild cognitive impairment and loss of functioning [3]. While ophthalmological symptoms such as optic atrophy, chorioretinitis, macular degeneration, and papilledema are commonly associated with SSPE, they usually occur concurrently with the neurological symptoms [2, 17]. This presentation of vision loss preceding neurological symptoms, while being previously documented, is rare [17]. 

The patient's brain MR demonstrated progressive cerebral atrophy, which is characteristically seen in imaging as clinical progression of the disease occurs [6]. The patient's imaging is similar to other Stage 2 patients described in a previous study showing a signal change in deep white matter, basal ganglia, and frontal and parietooccipital areas that can be normal in Stage 1 [6, 19]. 

The patient received standard treatments for SSPE, including isoprinosine, ribavirin, and interferon alpha. For treatment, alpha interferon has rarely been shown to recover loss of function, but more commonly it stabilizes the disease up to several years [7]. However, no notable improvement was seen in this case. 

In this case, treatment with carbamazepine resulted in dramatic, although temporary, symptomatic improvement in myoclonus. Combination therapy of carbamazepine and valproic acid was less effective than carbamazepine monotherapy, possibly due to autoinduction of the two antiepileptic medications that minimized the efficacy. Few case reports have shown a similar pattern of improvement of myoclonus with carbamazepine monotherapy, with the results summarized in Table 1 [6–12]. In two of these studies, carbamazepine initially improved myoclonus, then was discontinued, resulting in deterioration, and then was restarted, resulting in improvement [12, 15]. On the other hand, there are also case reports showing that carbamazepine is refractory to myoclonus [17, 18] with one report showing improvement with topiramate instead [18]. More recently, levetiracetam has been shown to improve both myoclonus and encephalopathy in a single patient with SSPE [20]. 

The use of carbamazepine in the symptomatic treatment of myoclonus seems counterintuitive. Carbamazepine has been previously shown to exacerbate a variety of seizure types (absence, atonic, and myoclonic) in patients with generalized epilepsies [21]. Therefore, one might expect that carbamazepine might precipitate worsening myoclonus in the light of periodic generalized epileptiform discharges on EEG. The exact mechanism of action of carbamazepine in SSPE is not entirely clear. The etiology for the myoclonus is thought to be due to basal ganglia involvement of the virus [4], whose pathways are not generally considered to be governed by alterations in sodium channels. While the patient's clinical symptomatology improved following carbamazepine, the MRI findings showed persistent hyperintensity at the level of the basal ganglia and progressive atrophy as has been previously reported [22]. 

The significant improvement of cognitive function after carbamazepine has been previously documented [13]. The simultaneous improvement of cognition and myoclonus has also been reported in a single patient treated with levetiracetam [20]. This may suggest a similar mechanism producing both the myoclonus and encephalopathy which needs to be further elucidated and is not consistent with sodium channel alterations based on our current understanding of the mechanism of action of levetiracetam. As our patient was symptomatic for nearly a year prior to the administration of carbamazepine, it remains to be seen if carbamazepine could have altered the course of the disease if earlier therapy had been initiated. 

The improvement seen in myoclonus following carbamazepine was not dose dependent. The results from our case indicate that carbamazepine, while being useful for providing temporary relief of symptoms, did not alter the pathophysiology of the disease. While the case reports and review of the literature demonstrate a trend in the improvement of symptoms in patients with SSPE, a randomized controlled trial is essential both to demonstrate significant benefit from carbamazepine and to justify the use of carbamazepine as a potential first line symptomatic treatment for SSPE. 

## Figures and Tables

**Table 1:** Summary of case series documenting use of carbamazepine therapy in the treatment of SSPE.

Number of patients	Patient age (years)	Case description summary	Reference
3	13, 13, 9	All 3 patients showed dramatic improvement in myoclonus that was temporary. One patient discontinued therapy because of carbamazepine allergy	[13]
1	26	Temporary improvement of myoclonus, followed by deterioration and death	[14]
1	17	Dramatic improvement of myoclonus, worsened on discontinuation of carbamazepine	[15]
1	30	Temporary improvement with carbamazepine	[16]
1	20	Dramatic improvement of myoclonus that worsened upon carbamazepine discontinuation	[12]
1	9	No response to carbamazepine therapy	[17]
1	4	No improvement on carbamazepine, improvement with topiramate	[18]
